# Impacts of Changes in Atmospheric O_2_ on Human Physiology. Is There a Basis for Concern?

**DOI:** 10.3389/fphys.2021.571137

**Published:** 2021-03-02

**Authors:** Ralph F. Keeling, Frank L. Powell, Gary Shaffer, Peter A. Robbins, Tatum S. Simonson

**Affiliations:** ^1^Scripps Institution of Oceanography, University of California, San Diego, La Jolla, CA, United States; ^2^Division of Pulmonary, Critical Care and Sleep Medicine, Department of Medicine, University of California, San Diego, La Jolla, CA, United States; ^3^GAIA Antarctic Research Center, University of Magallanes, Punta Arenas, Chile; ^4^Niels Bohr Institute, University of Copenhagen, Copenhagen, Denmark; ^5^Department of Physiology, Anatomy, and Genetics, University of Oxford, Oxford, United Kingdom

**Keywords:** atmospheric oxygen, fossil fuels, global change, evolution, V.O 2max, human health, hypoxia, high altitude

## Abstract

Concern is often voiced over the ongoing loss of atmospheric O_2_. This loss, which is caused by fossil-fuel burning but also influenced by other processes, is likely to continue at least for the next few centuries. We argue that this loss is quite well understood, and the eventual decrease is bounded by the fossil-fuel resource base. Because the atmospheric O_2_ reservoir is so large, the predicted relative drop in O_2_ is very small even for extreme scenarios of future fossil-fuel usage which produce increases in atmospheric CO_2_ sufficient to cause catastrophic climate changes. At sea level, the ultimate drop in oxygen partial pressure will be less than 2.5 mm Hg out of a baseline of 159 mmHg. The drop by year 2300 is likely to be between 0.5 and 1.3 mmHg. The implications for normal human health is negligible because respiratory O_2_ consumption in healthy individuals is only weakly dependent on ambient partial pressure, especially at sea level. The impacts on top athlete performance, on disease, on reproduction, and on cognition, will also be very small. For people living at higher elevations, the implications of this loss will be even smaller, because of a counteracting increase in barometric pressure at higher elevations due to global warming.

## Introduction

Direct observations since 1989 confirm that the atmospheric O_2_ abundance has been decreasing steadily year by year (Keeling and Manning, 2014). The O_2_ loss is the flip side of the CO_2_ buildup from fossil-fuel burning, and is expected to continue throughout the fossil-fuel era. The CO_2_ buildup is a major environmental concern, with consequences for global climate via the “greenhouse effect,” for land plants via “CO_2_ fertilization,” and for marine organisms via “ocean acidification” (Ciais et al., 2013). In comparison to the CO_2_ buildup, the O_2_ loss is very small in relative terms. CO_2_ has now risen from a preindustrial level of ∼277 ppm to a level of 410 ppm in year 2020 (Friedlingstein et al., 2020). The measured O_2_ loss has been of comparable magnitude in moles, but this dwarfed by the massive atmospheric store of O_2_, which comprises 21% of air. Still, the fact that O_2_ is measurably in decline raises concerns. Considering that O_2_ is essential for aerobic life, how sure are we that the continuing O_2_ decline won’t eventually have significant impacts?

We are aware of two prior reviews of this topic. The first, by Broecker (1970), makes a compelling case that the projected future O_2_ changes would be very small and likely insignificant. The second, by Martin et al. (2017), uses projections of much larger future O_2_ loss based on a parabolic model of Livina et al. (2015). Martin et al. (2017) systematically considered the major factors determining the potential impact of atmospheric oxygen (O_2_) depletion on human survival. They discussed the different time domains of effects of hypoxia, from acute responses, such as increased breathing and circulation, to longer-term physiological and cellular acclimatization, such as increased blood-O_2_ carrying capacity, and ultimately evolutionary genetic adaptations that increase reproductive success in high altitude populations. They also considered the range of responses, from relatively benign conditions such as acute mountain sickness to loss of consciousness and ultimately extinction. However, as we discuss below, the larger projected O_2_ losses from Livina et al. (2015) do not have a sound geochemical basis.

The purpose of this article is to reassess possible future O_2_ loss to address its possible importance for human physiology and health. We begin by reviewing the geochemical controls on O_2_ discussing likely magnitudes of changes and offering a critique of the Livina et al. (2015) prediction. Second, we provide scenarios for possible O_2_ trends over the next 1,000 years, accounting for impacts on O_2_ from fossil-fuel usage, land-use, warming, rising CO_2_, and changes in barometric pressure. At high elevations, global warming is predicted to increase barometric pressure (Moore and Semple, 2009), an effect which offsets the impact of O_2_ loss on the O_2_ partial pressure and which dominates above 3,000 m. To assess whether these O_2_ changes have potential physiological consequences, we then review the original research literature on the effects of hypoxia on human physiology. In addition to general physiological considerations, we discuss specific impacts on athletic performance, disease, altitude effects, reproduction, and evolution. In essence, we find that the physiological effects are too small to be of concern.

This article does not address a parallel question of whether the rising atmospheric CO_2_ might also have direct physiological impacts. This topic has been broached recently in studies focusing on cognitive impacts in indoor settings. Interested readers should consult Karnauskas et al. (2020) and references therein.

## Geochemical Context

We first briefly discuss units for O_2_ (see also Appendix A). The current atmospheric O_2_ inventory corresponds to 37125 Pmol O_2_ (1 Pmol = 10^15^ mol). A common unit for physiological studies is the O_2_ partial pressure that would be obtained if the air were fully dried at the same total pressure, which we call ${\text{P}}_{O_{2}}^{\prime }$. (The presence of water vapor reduces the actual O_2_ partial pressure slightly below ${\text{P}}_{O_{2}}^{\prime }$). ${\text{P}}_{O_{2}}^{\prime }$ is typically reported in mm of mercury (mmHg) (1 atm = 1013.25mb = 760.0 mm Hg) yielding a typical sea level value of 0.2094 × 760 = 159.1 mmHg. A further unit is changes in O_2_/N_2_ mole ratio, the conventional basis for reporting measured atmospheric O_2_ changes. These units are physically distinct but can be approximately related by simple scaling as discussed in Appendices A,B.

**FIGURE 1:**
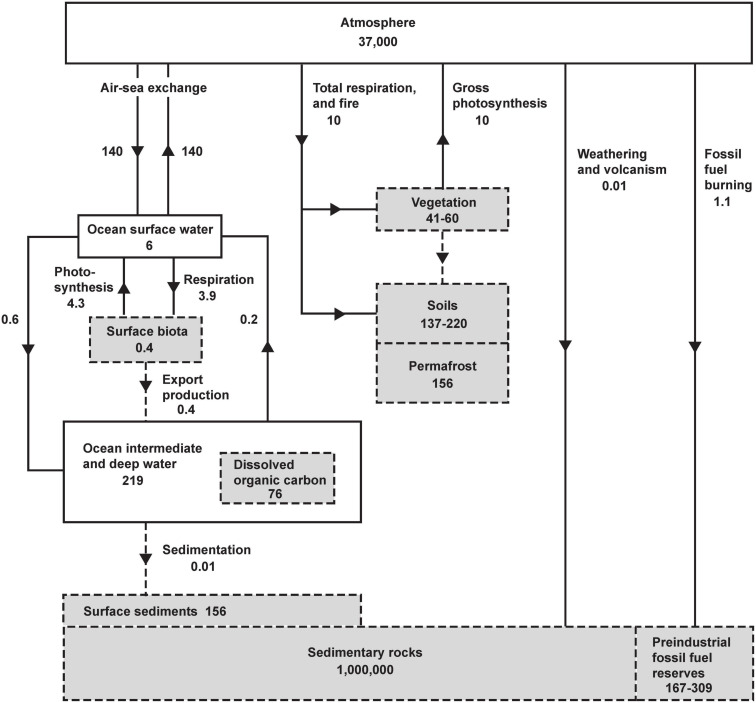
The global oxygen cycle, from Keeling (1988), showing short-term and long-term sources and sinks and coupling with the reservoirs of organic carbon in units of 10^15^ moles and 10^15^ moles year^– 1^. Oxygen fluxes and reservoirs are denoted by solid lines and solid boxes. Organic fluxes reservoirs are denoted by gray boxes with dashed perimeter, and organic fluxes with dashed lines. Organic matter is expressed in terms of O_2_ equivalent, i.e., the amount of O_2_ consumed when the material is fully oxidized. Organic reservoirs other than surface biota and sedimentary rocks have been updated using recent estimates from Ciais et al. (2013) using O_2_/C oxidative ratios of 1.1 (vegetation, soils, permafrost), 1.3 (dissolved organic carbon), and for fossil-fuel by fuel type from Keeling (1988). Fluxes and reservoirs other than fossil-fuel burning are notionally for a pre-industrial steady state. Fossil-fuel burning is for year 2019 (Friedlingstein et al., 2020).

The main controls on atmospheric O_2_ are shown inFigure 1. Gains or losses of atmospheric O_2_ are tied to gains or losses of dissolved O_2_ in the oceans or gains or losses of carbon in organic reservoirs. The chemistry of photosynthesis CO_2_ + H_2_O →CH_2_O + O_2_ produces O_2_ and organic carbon (represented schematically as CH_2_O). Both on land and in the oceans, virtually all organic matter produced by photosynthesis is eventually decomposed via the reverse reaction CH_2_O + O_2_ → CO_2_ + H_2_O. The full cycle of life is therefore a do-nothing loop with respect to O_2_ production, as the O_2_ produced during photosynthetic production of organic matter is offset by O_2_ consumed during its eventual decomposition. The stability of atmospheric O_2_ therefore hinges the stability of the organic carbon reservoirs rather than on gross rates of photosynthesis and respiration. As shown in Figure 1, however, the reservoirs of organic carbon on land and in the ocean, such as vegetation, soils, permafrost, and dissolved organic matter, and the reservoir of dissolved O_2_ in the ocean are all very small when compared to the massive atmospheric O_2_ reservoir. For example, even if all photosynthesis were to cease while the decomposition continued, eventually oxidizing all tissues in vegetation and soils, including permafrost, this would consume 435 Pmol, equivalent to a 1.9 mm Hg (1.2%) drop in ${\text{P}}_{O_{2}}^{\prime }$ at sea level. Although land and marine biota can impact O_2_ at small detectible levels, they are not the “lungs of the planet” in the sense of ensuring global O_2_ supply. Similarly, wildfire does not threaten the O_2_ supply, not just because fire is usually followed by regrowth, but also because the impact is bounded by limited pool of carbon in vegetation. These issues are widely misunderstood in popular science.

Earth’s O_2_ supply is actually a massive geologic deposit, stored in the atmosphere rather than the solid earth and closely tied to organic matter stored in sedimentary rocks (Royer, 2014). While this deposit is a distant byproduct of photosynthesis, its size is controlled by geological processes: (1) the incorporation of organic detritus into newly forming sea-floor sediments isolated from the atmosphere, and (2) by the uplift and exposure ancient sediments and volcanic gases to atmospheric oxidation. These are very slow processes, which are capable of producing significant changes in atmospheric O_2_ only on time scales of millions of years (Berner, 1999). These processes also control the global amount of organic carbon stored in sedimentary formations, such as shales. Very little of this carbon has economic value, but a small component is exploitable as fossil-fuels.

At current rates, the extraction and burning of fossil fuels is equivalent to at least a 100-fold acceleration of the global exposure process, which thereby dominates humanity’s small impact on atmospheric O_2_ (Shaffer et al., 2008). The estimated fossil-fuel reserves in Figure 1 are based on Ciais et al. (2013). The upper estimate corresponds to an equivalent O_2_ loss of ∼309 Pmol or a drop of 1.3 mm Hg in ${\text{P}}_{O_{2}}^{\prime }$ from the preindustrial level. This would be achieved in ∼200 years if consumption continues at the current rate. This high estimate is not a categorically upper bound, however. If large unconventional and undiscovered fossil energy resources are ultimately exploited, an even larger decline in O_2_ is possible. Taking an estimate of ∼5,000 Pg C for the ultimate resource of fossil-fuels (Rogner, 1997) and assuming an oxidative ratio of 1.4 for fossil-fuels yields an upper bound on O_2_ loss of 580 Pmol O_2_ or a ${\text{P}}_{O_{2}}^{\prime }$ drop of 2.5 mmHg.

**FIGURE 2:**
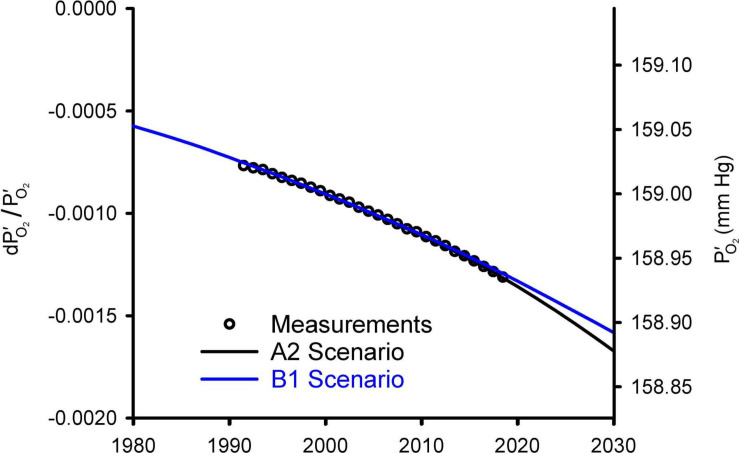
Modeled changes in atmospheric O_2_ from Shaffer et al. (2009) versus observed global averages from the Scripps O_2_ program (Keeling and Manning, 2014). The O_2_ levels are shown on left as fractional change in O_2_ partial pressure relative to a preindustrial reference and on right as absolute partial pressure. The observations, originally reported as changes in O_2_/N_2_ ratio in per meg units (see Appendix A), were converted to dP′_O_2_/${\text{P}}_{O_{2}}^{\prime }$ and offset with an additive constant to align with the model results. Global averages are based on the data from Alert (82.5°N), La Jolla (32.9°N), and Cape Grim (40.7°S) stations, following Keeling et al. (1996).

Figure 2 compares the observed changes in O_2_/N_2_ from 1991 to 2018 against simulations using a simple carbon/climate model by Shaffer et al. (2009) for the so-called A2 and B1 scenarios (Nakicenovic et al., 2000). These simulations account for O_2_ changes from fossil fuel burning and land use, which promotes decomposition of vegetation and soils, and account for the impact on ocean O_2_ and land carbon from CO_2_ fertilization and warming effects. Further details on these simulations are given in Appendix C. Without being explicitly tuned for O_2_ changes, the model for the A2 scenario accurately simulates the O_2_/N_2_ changes over the period of direct observations from 1991 to 2018. The success is partly attributable to the fact that fossil-fuel emissions from 1991 to 2018 have tracked the A2 scenario quite closely. The success also depends on quasi-realistic accounting for global influences on atmospheric O_2_ from land biospheric and ocean processes, which partly offset the O_2_ loss from fossil-fuel burning. The simulations are useful for documenting that the recent O_2_ changes are quite well understood. The observed drop in O_2_/N_2_ over 28 years from 1991 to 2018 period corresponds to 20 Pmol O_2_ or -0.09 mmHg. The model suggest that the full drop from before the industrial revolution to 2018 has been 48 Pmol or 0.21 mmHg.

So what should be made of the large predicted future O_2_ losses of Livina et al. (2015)? Their prediction is based on an extrapolation of parabolic fit to recent atmospheric observations, an approach which lacks a geochemical basis. The success of the model simulations in Figure 2 shows that there is no evidence of a major missing process justifying such an open-ended approach. The Livina et al. loss curve is equivalent to extrapolating fossil-fuel consumption into the future based on recent fuel-use trends without accounting for eventual resource limitations.

### Predicted Changes in Atmospheric O_2_ Over the Next 1,000 Years

**FIGURE 3:**
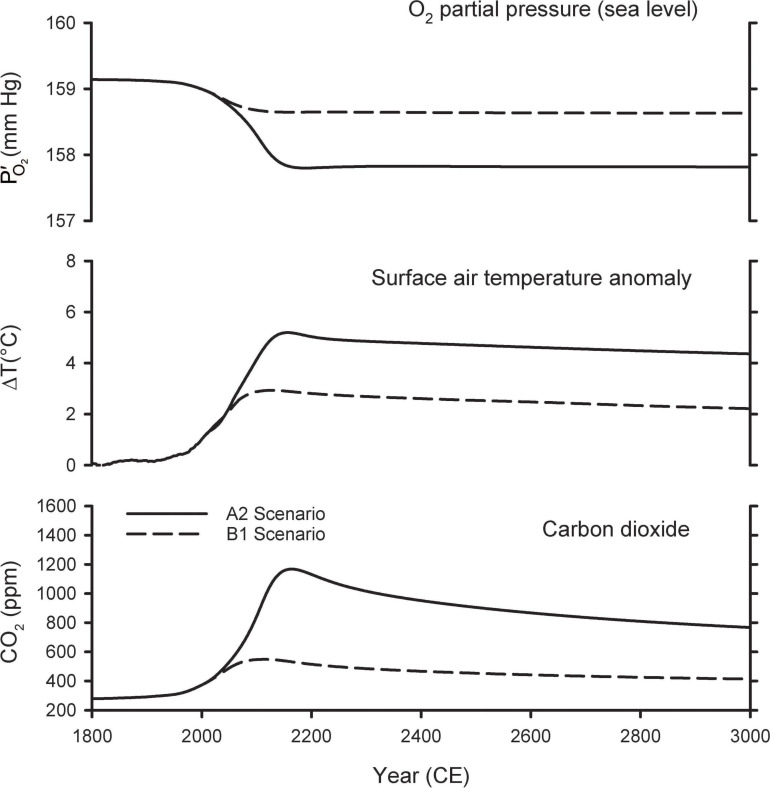
Predicted changes in atmospheric O_2_ partial pressure (${\text{P}}_{{\text{O}}_{2}}^{\prime }$), temperature, and CO_2_ mole fraction from model simulations of Shaffer et al. (2009).

Figure 3 shows the predictions of the A2 and B1 scenarios from the Shaffer et al. (2009) model extended out for the next 1,000 years. The A2 scenario is a high-end emissions scenario that assumes that fossil-fuel consumption is capped at 403 Pmol O_2_, which is slightly above the fossil-fuel reserves upper bound in Figure 1. The A2 scenario is similar to the more recent RCP8.5 scenario, which assumes an improbable fivefold expansion of coal use by 2100 and neglects competition from renewable energy sources, such as wind and solar (Hausfather and Peters, 2020). Warming in the A2 scenario peaks at 5.2°C, CO_2_ peaks at ∼1,200 ppm, and ${\text{P}}_{O_{2}}^{\prime }$ at sea level drops by 1.3 mmHg in year 2300. The B1 scenario is a middle-of-the-road scenario, allowing for modest mitigation effort, and assumes fossil-fuel consumption is capped at 159 Pmol O_2_. In B1, warming peaks at 2.9°C, CO_2_ peaks at ∼550 ppm, and ${\text{P}}_{O_{2}}^{\prime }$ at sea level drops by 0.50 mmHg in year 2300. In both scenarios, the O_2_ loss from fossil-fuel burning and land-use is partially offset by O_2_ release from the ocean due to warming and release from the land biosphere due to CO_2_ fertilization of plant growth. Without these offset, the O_2_ decline (from fossil-fuel and land use) would have amounted to 1.8 mmHg in the A2 scenario and 0.7 mmHg in the B1 scenario.

Shaffer et al. (2009) do not address changes in O_2_ partial pressure at higher altitudes, where barometric pressure is expected to increase due to upwards expansion of the atmosphere with warming (increasing scale height). In Table 1, we estimate the expected barometric pressure increase as a function of altitude corresponding to 3°C of surface warming for two cases, corresponding either to tropical or mid latitude atmospheric profiles. The calculations allow for uncertainty in the pressure increase associated with alternate assumptions for how the temperature lapse rate evolves, bracketed by assuming either that the lapse rate remains unchanged, or assuming it decreases in magnitude with warming as for a saturated moist adiabatic lapse rate. Current understanding suggests the lapse rate changes will lie between these limits (Bony et al., 2006). The differences between the tropical and mid latitude cases are small. In the following we consider only the tropical case.

**TABLE 1:** Barometric pressure increase at altitude driven by 3°C of surface warming.

	**Tropical**		**Mid latitude**	
**Initial temperature at sea level**	**25°C**		**15°C**	

**Constant lapse rate**	**4.5°C km^–1^**		**6°C km^–1^**	

**Altitude (km ASL)**	**Initial pressure (hPa)^a^**	**Pressure increase (hPa)^b^**	**Initial pressure(hPa)^a^**	**Pressure increase (hPa)^b^**

6	494.59	4.6 ± 0.9	475.34	5.0 ± 1.0
4	633.21	3.6 ± 0.6	618.99	3.9 ± 0.6
2	804.00	2.2 ± 0.3	796.13	2.2 ± 0.2
0	1031.25	0	1031.25	0

*^*a*^Calculated as the average of two separate pressure profiles ((P_*moist*_ + P_*const*_)/2), where P_*moist*_ assumes a moist adiabatic lapse rate and P_*const*_ assumes the specified constant lapse rate. The value of the constant lapse was selected so that P_*const*_ is similar to P_*moist*_ up to 6 km. Calculations done with formulae and constants from Jacobson et al. (2005). ^*b*^Pressure increase caused by 3°C of surface warming, computed by averaging the pressure changes expected for constant versus moist lapse rates: ΔP = (ΔP_*moist*_ + ΔP_*const*_)/2 whereΔP_*moist*_ = P_*moist*,_*_*f*_* – P_*moist*,_*_*i*_* and ΔP_*const*_ = P_*const*,_*_*f*_* – P_*const*,_*_*i*_* and where *i* and *f* denote initial and final profiles. The constant lapse rate is not changed between initial and final profiles, while the moist lapse rate changes as expected with temperature. The uncertainties are set equal to ± (ΔP_*moist*_ - ΔP_*const*_)/2. The moist lapse rate case yields the larger pressure increase, i.e. ΔP_*moist*_ > ΔP_*const*_.*

**FIGURE 4:**
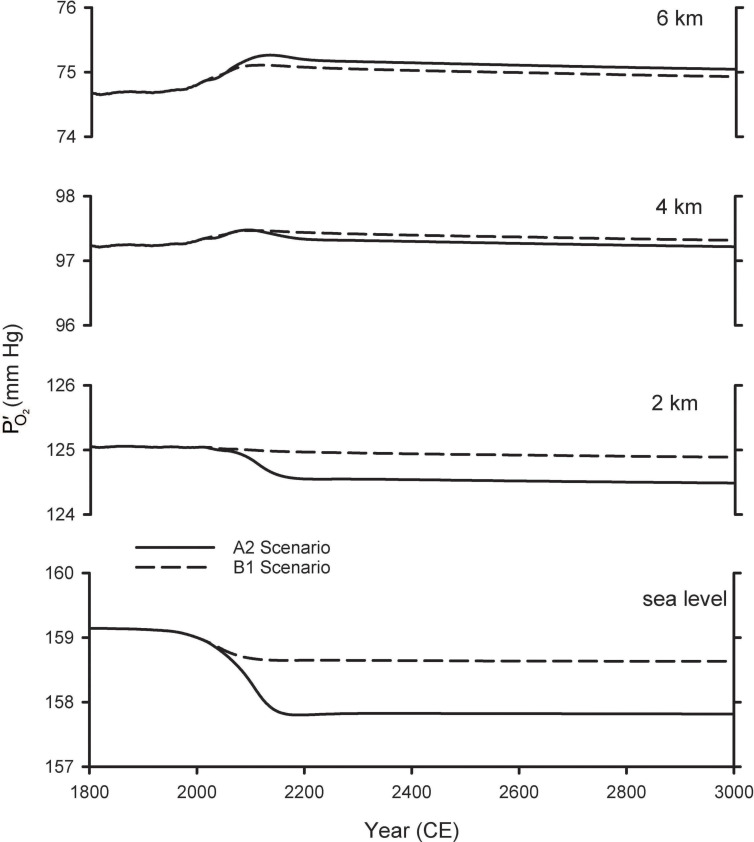
Predicted changes in O_2_ partial pressure (${\text{P}}_{O_{2}}^{\prime }$) at different elevations. The curves for sea level are from Shaffer et al. (2009) and repeated from Figure 2. The curves for other elevations were calculated by accounting both for global O_2_ loss and for changes in barometric pressure with warming (Table 1), scaled by the modeled temperature changes from Shaffer et al. (2009).

Figure 4 shows the resulting estimates for future variations in ${\text{P}}_{O_{2}}^{\prime }$ at various altitudes. These calculations account for changes in ${\text{P}}_{O_{2}}^{\prime }$ due both to changes in the global O_2_ inventory from Shaffer et al. (2009), as well as the changes in barometric pressure with altitude, assuming pressure increases linearly with surface warming using the central estimates for the tropics from Table 1. At 2 km above sea level (ASL) the predicted ${\text{P}}_{O_{2}}^{\prime }$ changes are considerably smaller than those at sea level, while at 4 and 6 km, the ${\text{P}}_{O_{2}}^{\prime }$ is predicted to increase as the barometric pressure change more than offsets the O_2_ losses. The cross-over altitude at which there is effectively no change in ${\text{P}}_{O_{2}}^{\prime }$ is 3.6 km for A2 and 2.5 km for B1.

## Evaluation of Physiological Consequences of the Predicted O_2_ Declines

At the outset, it is clear that any physiological effects of these O_2_ changes must be very small because the O_2_ changes under consideration are very small. The physiological effect of O_2_ is determined by the partial pressure, which depends on the product of the O_2_ concentration and total barometric pressure. Whether the O_2_ partial pressure changes from changing barometric pressure or O_2_ concentration, the physiological consequences are similar, especially for small O_2_ changes (Richalet, 2020). Table 2 summarizes the predicted changes in ${\text{P}}_{O_{2}}^{\prime }$ for year 2300 for the A1 and B1 scenarios, as well as the equivalence of these changes in terms of altitude shift. The equivalent altitude change is consistently 70 m or less over all altitudes of human habitation (<5 km). These changes in ${\text{P}}_{O_{2}}^{\prime }$ are similar in magnitude or smaller than the changes that occur during the passage of moderately strong storm systems. We are aware of no studies that have attempted to resolve physiological impacts of such tiny changes in P_O_2__, which would be in the noise range for physiological studies. Our analysis thus draws on inferences from studies over larger ranges in P_O_2__ and from theoretical considerations.

**TABLE 2:** Baseline values and changes in year 2,300 relative to baseline.

**Alt-itude**	**Baro-metric pressure**	**${\text{P}}_{O_{2}}^{\prime }$**	**${\dot{\text{V}}}_{{\text{O}}_{2\,m\,a\,x}}$**	**${\dot{\text{V}}}_{{\text{O}}_{2\,m\,a\,x}}$ slope**	**Equiv. altitude**		**Change in ${\text{P}}_{O_{2}}^{\prime }$**		**Change in**	
**(km ASL)**	**(mb)**	**(mmHg)**	**(% of sl value)**	**(% mmHg^–1^)^*a*^**	**change (m)**		**(mmHg)**		**${\dot{\text{V}}}_{{\text{O}}_{2\,m\,a\,x}}$ (%)**	
					**A2**	**B1**	**A2**	**B1**	**A2**	**B1**
6	494.59	74.7	66	0.89	–50	–38	+0.50	+0.39	+0.45	+0.35
4	633.21	97.2	80	0.46	–8	–16	+0.09	+0.20	+0.04	+0.09
2	804.00	125.0	93	0.27	32	6	–0.50	–0.09	–0.13	–0.02
0	1031.25	159.1	100	<0.27	70	26	–1.32	–0.50	(–0.4)^b^	(–0.1)^b^

*^*a*^For healthy untrained individual. Slope at sea level is unknown but very small. In elite athletes, a larger slope has been measured (see text). ^*b*^Upper bound.*

### Normal Healthy Individuals

To understand how changes in O_2_ may affect humans, we consider the basics of respiratory physiology for healthy humans (Guyton and Hall, 1996) using an approach similar to that of Martin et al. (2017) but focusing on much smaller decreases in P_O_2__ consistent with the geochemical analysis presented above. O_2_ is essential for life because it provides electrons to mitochondria, cellular organelles that use these electrons to generate the molecule ATP. ATP is vital as the primary fuel for cellular ion pumps that are necessary for nerve and brain function, muscle contraction and movement, the heart beat and breathing, digestion and metabolic functions, the synthesis of reproductive hormones and all other physiological functions necessary to sustain life.

**FIGURE 5:**
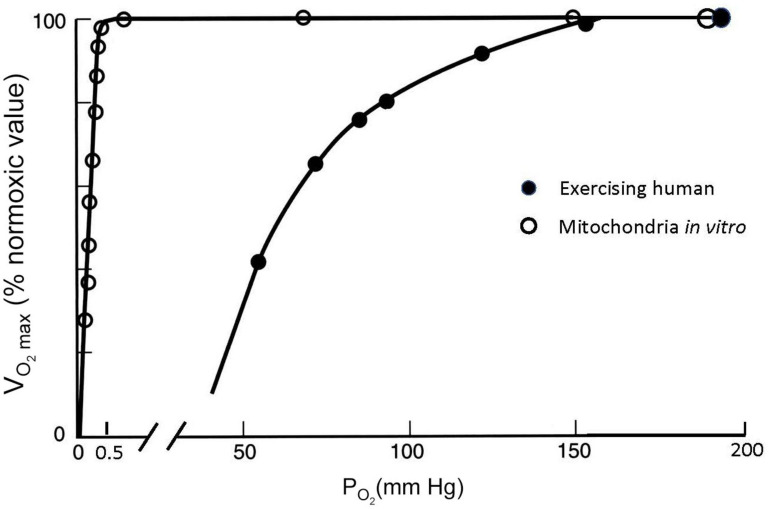
Maximal O_2_ consumption (V̇_O_2max_, as % of the maximum value at P_O_2__ = 150 mmHg) measured at different ambient P_O_2__ conditions for isolated mitochondria in a saline suspension (open symbols) and humans on a bicycle ergometer (closed symbols). V̇_O_2max_ decreases at inspired P_O_2__ levels in exercising humans much greater than those necessary to decrease V̇_O_2max_ in isolated mitochondria, which can be explained by P_O_2__ around the mitochondria in exercising humans falling to less than 1 mmHg (see text). After Gnaiger (2001) and Pugh et al. (1964).

Figure 5 shows how the cellular process of generating ATP depends on the amount of O_2_ available. Isolated mitochondria studied *in vitro* can maintain a maximum rate of ATP production and maximum O_2_ consumption, which is abbreviated as V̇_O_2max_, until O_2_ drops to very low values (Gnaiger, 2001). O_2_ levels are shown as partial pressure (P_O_2__) because this determines the “pressure head” driving diffusion for O_2_ into mitochondria, as well as O_2_ diffusion from the lungs into blood and from the blood into cells. The results shown in Figure 5 are obtained *in vitro* by adding metabolic substrates to a solution with mitochondria at high P_O_2__ levels until a maximum value of O_2_ consumption is measured, i.e., ${\dot{\text{V}}}_{{\text{O}}_{2}\,\max}$. Then the level of substrate is maintained while P_O_2__ is decreased. V̇_O_2max_ is expressed as the ratio (in percent) to the value of ${\dot{\text{V}}}_{{\text{O}}_{2}\,\max}$ at normal P_O_2__ values of 159 mmHg, known as “normoxia.” Figure 5 shows that no significant change in ${\dot{\text{V}}}_{{\text{O}}_{2}\,\max}$ observed in mitochondria until P_O_2__ drops below 1–2 mmHg; ${\dot{\text{V}}}_{{\text{O}}_{2}\,\max}$ decreases to 50% of the normoxic value when P_O_2__ is <0.5 mmHg. ${\dot{\text{V}}}_{{\text{O}}_{2}\,\max}$ is a reproducible and biologically meaningful way to quantify the effects of decreased P_O_2__ on physiological processes.

The effects of decreasing P_O_2__ on ${\dot{\text{V}}}_{{\text{O}}_{2}\,\max}$ are very different, however, when they are measured in humans doing exercise as also illustrated in Figure 5. For this experiment data, a person is asked to pedal an exercise bike as hard as possible and the resistance or work load is increased while the subject’s O_2_ consumption is measured (with gas analyzers connected to a mouthpiece used to measure ventilation, Pugh et al., 1964). At some point, O_2_ consumption will reach a plateau as the work rate increases. This point defines the ${\dot{\text{V}}}_{{\text{O}}_{2}\,\max}$ (any additional work possible beyond this is powered by anaerobic metabolism, e.g., from lactic acid). The same can be repeated at different levels of ambient P_O_2__, establishing a curve of ${\dot{\text{V}}}_{{\text{O}}_{2}\,\max}$ as a function of ambient P_O_2__. In most healthy subjects (though not highly trained athletes as discussed below), the measured ${\dot{\text{V}}}_{{\text{O}}_{2}\,\max}$ plateaus when P_O_2__ approaches a normal sea level value of around 159 mmHg. However, ${\dot{\text{V}}}_{{\text{O}}_{2}\,\max}$ decreases dramatically with modest decreases in P_O_2__, in stark contrast to the isolated mitochondria (Figure 5). V̇_O_2max_ falls to half the value measured at sea level when inspired P_O_2__ is dropped to 60 mmHg. The isolated mitochondria experiment demonstrated that basic cellular function is *not* affected by such modest decreases in P_O_2__ so something is different in whole organisms that makes us more sensitive to decreased P_O_2__, i.e., hypoxia.

**FIGURE 6:**
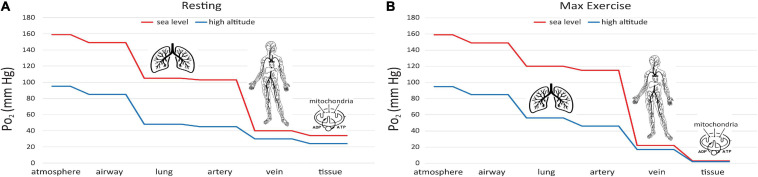
Oxygen cascade, showing how P_O_2__ decreases from the atmosphere to mitochondria along the physiological O_2_ transport chain at sea level (red) and high altitude (blue). **(A)** person at rest, **(B)** person at maximum exercise.

This difference can be understood via the “oxygen cascade,” which quantifies the fall in P_O_2__ at sequential steps of physiological transport of O_2_ from the atmosphere to the mitochondria. Figure 6A shows this cascade using values typical of a healthy subject at rest (Richardson et al., 2006). Two curves are shown, one for sea level and one for 1.5 km ASL. At the first step, P_O_2__ decreases when air is inspired from the atmosphere into the nose and upper airways. The decrease occurs because the air gains water vapor, as it saturates at a normal body temperature of 37°C, yielding a water vapor pressure 47 mmHg. The P_O_2__ in the airway is designated P_IO_2_. At current O_2_ levels, P_IO_2_ is 149 mmHg at sea level and 123 mmHg at 1.5 km ASL. Because the humidity in the airway is fixed by body temperature, the inspired P_O_2__ level is independent of ambient humidity. In contrast, the P_O_2__ in the atmosphere will be higher if the air is drier. A suitable measure of ambient O_2_ which determines P_IO_2_ is the partial pressure that would be obtained in perfectly dry air at ambient pressure, the quantity defined in the previous section as ${\text{P}}_{O_{2}}^{\prime }$. The drop in P_O_2__ from dry ambient air to inspired air is ∼10 mmHg whether at sea level or 1.5 km ASL. The humidification effect is relatively larger at high altitude since it remains constant at 10 mmHg regardless of atmospheric pressure and temperature; e.g. on the summit of Mt. Everest where barometric pressure is only 1/3 that at sea level (West, 1996), P_O_2__ decreases almost 20% with humidification versus 6% at sea level.

A significant decrease in P_O_2__ occurs at the step between inspired P_O_2__ in the airways and the alveoli in the lungs where O_2_ exchange with blood occurs (Figure 6A). The magnitude of this decrease depends on the efficiency of convective (bulk) transport of air into the lungs by ventilation. The next step occurs as O_2_ diffuses from the alveoli into capillary blood in the lungs and is pumped by the heart to the tissues in arterial blood. The decrease between alveolar and arterial P_O_2__ is very small, at least in healthy lungs that have an excess of diffusing capacity under resting conditions. There are some small differences in P_O_2__ to this point in the oxygen cascade at moderately high altitude versus sea level, since breathing rate increases at high altitude to maintain alveolar P_O_2__ near normal levels, at least under resting conditions.

**FIGURE 7:**
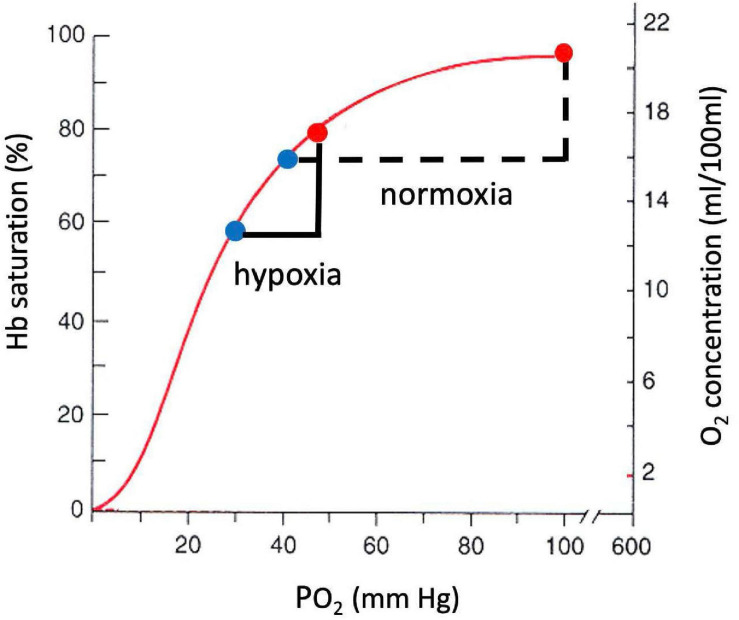
O_2_-hemoglobin equilibrium curve plotting the % hemoglobin saturation with O_2_ (left) or O_2_ concentration in blood (right) versus P_O_2__. The sigmoidal shape of the curve allows a similar change in O_2_ concentration for a smaller change in P_O_2__ in hypoxia versus normoxia.

A large decrease in P_O_2__ occurs also along the oxygen cascade between arterial and venous P_O_2__. The amount of O_2_ transported by blood flow is a non-linear function of the P_O_2__ of the blood, rising rapidly at low P_O_2__ but then saturating at higher P_O_2__. The relationship is explained by O_2_-hemoglobin equilibrium, curve shown in Figure 7, which relates P_O_2__ to the actual O_2_ concentration [O_2_], or the O_2_ saturation, S_O_2_, of hemoglobin available in the blood (Guyton and Hall, 1996). S_O_2_ is conveniently measured with a finger pulse oximeter and is the most widely used measure of arterial oxygenation. Of critical importance is the arterial to venous difference in [O_2_] which represents the amount of O_2_ that diffused out of capillaries to be consumed by the mitochondria. This difference depends on (1) the rate of O_2_ consumption by the tissues and (2) the blood flow to the tissues, e.g., cardiac output. Since the O_2_ demand and cardiac output are similar at rest at sea level and high altitude, the arterial-venous difference in [O_2_] is similar. Due to the non-linear shape of the O_2_-hemoglobin equilibrium curve, this translates into larger differences in P_O_2__ at sea level versus altitude. As long as venous P_O_2__ remains above a few mm Hg, as required by the mitochondrial V̇_O_2max_ curve (cf. Figure 5), this is sufficient to satisfy mitochondrial demand.

It is also instructive to consider cases of increased O_2_ demand, which are illustrated in Fig. 6B. showing the effects of exercise on the oxygen cascade at sea level and high altitude (Richardson et al., 2006). As O_2_ consumption increases, alveolar P_O_2__ can actually increase from hyperventilation, especially at high altitude where there is already a reflex increase in ventilation to counteract decreased O_2_ supply. The decrease from alveolar to arterial P_O_2__ can increase with exercise too, especially at high altitude where diffusion may not be adequate even in healthy lungs to support complete equilibration in P_O_2__ between the alveoli and pulmonary capillaries. Also, exercise generally decreases venous P_O_2__ because cardiac output cannot increase as much as O_2_ consumption (5–7 fold versus 10–20 fold, Guyton and Hall, 1996). Finally, increased O_2_ demand requires greater diffusion of O_2_ across the capillaries into the mitochondria. Since the diffusion rate is proportional to the gradient in P_O_2__, increased demand therefore produces an even larger drop in P_O_2__ in the mitochondria than in the venous blood at the end of the capillaries. The ultimate limit on O_2_ demand is set at the point at which P_O_2__ in the mitochondria drops below ∼1 mmHg, thus limiting ATP synthesis as for mitochondria *in vitro* (Figure 5). In principle, this limit (or equivalently V̇_O_2max_) is sensitive to all of the steps in the O_2_ cascade including ambient ${\text{P}}_{O_{2}}^{\prime }$ levels (Wagner, 1996).

Of critical importance to assessing the impacts of declining atmospheric O_2_ on normal human metabolism is the plateau of the ${\dot{\text{V}}}_{{\text{O}}_{2}\,\max}$ curve near normal sea level ${\text{P}}_{O_{2}}^{\prime }$ levels. This plateau implies that small changes in ${\text{P}}_{O_{2}}^{\prime }$ can have only a very small impact on maximum O_2_ uptake rates. Although ${\dot{\text{V}}}_{{\text{O}}_{2}\,\max}$ is ultimately determined by the combined interaction of all steps in the oxygen cascade, the existence of the plateau depends on normal humans having S_O_2_ close to 100%, where the S_O_2_ is almost independent of arterial ${\text{P}}_{O_{2}}^{\prime }$(Wagner, 1996). This helps buffer arterial O_2_ supply from upstream changes in lung function or ambient ${\text{P}}_{O_{2}}^{\prime }$.

We are not aware of measurements of slope of the ${\dot{\text{V}}}_{{\text{O}}_{2}\,\max}$ curve for healthy individuals at sea level, as this slope is in any case very small. In Table 2, we adopt as an upper bound a value corresponding to the slope at 2 km, which together with the estimated change in ${\text{P}}_{O_{2}}^{\prime }$ from the A2 scenario in year 2300 yields an estimated change in ${\dot{\text{V}}}_{{\text{O}}_{2}\,\max}$ of -0.4%. A best guess might be between -0.1 and -0.2%, taking a more reasonable estimate of the eventual O_2_ loss and a lower slope of the V̇_O_2max_ curve. We note that a slightly larger change in ${\dot{\text{V}}}_{{\text{O}}_{2}\,\max}$ is expected for elite athletes, who are more sensitive to changes in ${\text{P}}_{O_{2}}^{\prime }$, as discussed below. However, the decrease in P’_O_2_ would have to be an order of magnitude greater than the ∼1.3 mm Hg predicted to have a significant effect on performance, if we consider a 10% reduction in ${\dot{\text{V}}}_{{\text{O}}_{2}\,\max}$ is not observed until P_O_2__ drops to 125 mm Hg in healthy individuals at sea level (Figure 5).

The ${\dot{\text{V}}}_{{\text{O}}_{2}\,\max}$ curve as shown in Figure 5 applies to an individual exposed to short-term changes in P_O_2__. For long-term exposure, the responses are typically smaller because of physiological acclimatization, whereby the body increases its capacity to uptake and deliver O_2_. Acclimatization has been observed, not just for large changes in P_O_2__, but also for quite small changes (Donoghue et al., 2005). This is yet another reason why the changes in ${\dot{\text{V}}}_{{\text{O}}_{2}\,\max}$ driven by falling ${\text{P}}_{O_{2}}^{\prime }$ are likely to be very small for a healthy individual.

### Highly Trained Athletes

Highly trained athletes are likely more sensitive to changes in O_2_ levels than most people. For example, elite athletes actually show decreased arterial O_2_ saturation during maximum exercise at sea level because they have extremely high cardiac outputs and high pulmonary blood flow so there is insufficient time for O_2_ equilibration in the lungs, resulting in a diffusion impairment. Consequently mild *in*creases in O_2_ (5% inspired, PIO_2_ = 149–185 mmHg) significantly increases ${\dot{\text{V}}}_{{\text{O}}_{2}\,\max}$ in trained athletes with an average ${\dot{\text{V}}}_{{\text{O}}_{2}\,\max}$ = 70 ml O_2_/kg/min but not in normal subjects with an average ${\dot{\text{V}}}_{{\text{O}}_{2}\,\max}$ = 57 ml O_2_/kg/min (Powers et al., 1989). Such athletes do not show as pronounced a plateau in whole body ${\dot{\text{V}}}_{{\text{O}}_{2}\,\max}$ versus P_O_2__ at normal sea level P_O_2__ as indicated in Figure 5. Trained athletes have both increased mitochondria and capillary surface area for increased O_2_ diffusion in tissues, in addition to increased O_2_ transport at other stages along the oxygen cascade, to support this increased O_2_ consumption.

In a study with trained athletes, Wehrlin and Hallén (2006) detected a decrease in ${\dot{\text{V}}}_{{\text{O}}_{2}\,\max}$ for an increase in altitude from only 300 to 800 m ASL (Wehrlin and Hallén, 2006) associated with an 8 mmHg decrease in inspired P_O_2__ (144–136 mm Hg). The change was consistent with measurements over a larger altitude range showing a decrease of 6.3% in ${\dot{\text{V}}}_{{\text{O}}_{2}\,\max}$ for every 1 km increase in altitude ASL (Wehrlin and Hallén, 2006), corresponding to a slope of ∼0.37% per mmHg. On this basis, if ${\text{P}}_{O_{2}}^{\prime }$ at sea level drops by 2 mmHg, as might occur in an extreme scenario, this would yield a decrease of 0.75% in ${\dot{\text{V}}}_{{\text{O}}_{2}\,\max}$. The more likely scenario assuming 0.5 mmHg decrease would yield a 0.18% drop in ${\dot{\text{V}}}_{{\text{O}}_{2}\,\max}$. This study used subjects with ${\dot{\text{V}}}_{{\text{O}}_{2}\,\max}$ of ∼ 66 ml O_2_/kg/min (Wehrlin and Hallén, 2006) compared to a normal value for a healthy individual of ∼40 ml O_2_/kg/min. Other reviews of the literature have found linear decreases in ${\dot{\text{V}}}_{{\text{O}}_{2}\,\max}$ with inspired P_O_2__ between 150 and 80 mmHg and predict a similar decrease in ${\dot{\text{V}}}_{{\text{O}}_{2}\,\max}$ of less than 1% for the 1.5 mmHg decrease (Gonzalez and Kuwahira, 2018).

Athletic performance in aerobic sports such as long distance running has been shown to correlate with ${\dot{\text{V}}}_{{\text{O}}_{2}\,\max}$ when comparing individuals with large differences in ${\dot{\text{V}}}_{{\text{O}}_{2}\,\max}$ (Bassett and Howley, 2000). On this basis it is conceivable that a 0.5 mm Hg decrease in ${\text{P}}_{O_{2}}^{\prime }$ due to global O_2_ loss could cause a 0.18% drop in ${\dot{\text{V}}}_{{\text{O}}_{2}\,\max}$ in elite athletes, and this in turn could cause a ∼0.18% increase in e.g., marathon times. But this extrapolation is fraught with uncertainty. Establishing a causal connection between small (e.g., <1%) changes in ${\dot{\text{V}}}_{{\text{O}}_{2}\,\max}$ and performance is very difficult because other metabolic factors, such as lactic acid accumulation and running economy, dominate over such small ranges (Bassett and Howley, 2000). It thus seems very unlikely that the small conceivable change in performance due to dropping ${\text{P}}_{O_{2}}^{\prime }$, occurring over centuries, could be detected in the face of changes in training, nutrition, talent recruitment, genetic changes, doping, etc.

### High Altitude

The non-linearity of the ${\dot{\text{V}}}_{{\text{O}}_{2}\,\max}$ curve implies greater sensitivity to P_O_2__ changes at altitude. However, the modeling of atmospheric changes discussed earlier produces an interesting result that ${\text{P}}_{O_{2}}^{\prime }$ may actually *in*crease above 3,000 m in 2300 CE versus today due to increasing barometric pressure with warming. One way to assess these competing effects is to calculate the expected changes ${\dot{\text{V}}}_{{\text{O}}_{2}\,\max}$ at different altitudes, accounting the predicted changes in P_O_2__ and the non-linearity of the ${\dot{\text{V}}}_{{\text{O}}_{2}\,\max}$ curve. Table 2 summarizes this calculation for year 2300 at sea level, 2, 4, and 6 km ASL. At 2 km and below, ${\dot{\text{V}}}_{{\text{O}}_{2}\,\max}$ is calculated to decline, but the magnitude of the changes is extremely small, less than 0.4% for both A2 and B1 scenarios. At 4 and 6 km ${\dot{\text{V}}}_{{\text{O}}_{2}\,\max}$ is calculated to increase, with larger gains at the highest altitudes. Not only are these changes very small over all altitudes of normal human habitation <5 km ASL (West, 2002), but they also neglect the possibility of acclimatization, which would reduce impacts further. The calculated changes apply to an individual, living at sea level and traveling to higher altitude, who has NOT acclimatized to the small ${\text{P}}_{O_{2}}^{\prime }$ changes at sea level. The impact on ${\dot{\text{V}}}_{{\text{O}}_{2}\,\max}$ at altitude after any such acclimatization would necessarily be even smaller.

Even greater gains in P_O_2__ and ${\dot{\text{V}}}_{{\text{O}}_{2}\,\max}$ can be expected above 6 km. As discussed by Moore and Semple (2009), this may have benefits for high altitude mountaineering, although Moore and Semple (2009) slightly overestimate the benefits because they consider only the impact of changes in barometric pressure and not the impact of global O_2_ loss.

It is interesting that the reversal of the direction of change in P_O_2__ and ${\dot{\text{V}}}_{{\text{O}}_{2}\,\max}$ versus altitude with global change occurs very near the threshold for acute mountain sickness (AMS) in today’s atmosphere. AMS symptoms include headache, insomnia, lassitude, anorexia, and/or nausea that occur above 3,000 m in most subjects, but can occur in the most sensitive individuals with rapid ascent to only 2,400 m (Hultgren, 1997). Hence, AMS could occur in the most sensitive subjects at lower altitude in the future while less sensitive subjects may be better able to tolerate higher altitude, i.e., the altitude range over which AMS symptoms appear could be expanded with global change. The altitude threshold changes are very small in any case, e.g., <50 m. Similar predictions can be made for more serious altitude illnesses such as high altitude pulmonary or cerebral edema that are extremely rare with even rapid ascent to altitude less than 2,400 or 2,700 m, respectively (Hultgren, 1997).

### Disease

Lung disease reduces the efficiency of O_2_ uptake causing arterial P_O_2__ levels to be lower in patients compared to normal subjects for a given inspired P_O_2__. Such hypoxemia has a major impact on many physiological functions and provides the rationale for supplemental oxygen therapy in patients with chronic heart and lung disease. The American College of Physicians (ACP) recommends supplemental long-term oxygen therapy in all patients who have severe resting hypoxemia, defined as an arterial P_O_2__≤59–55 mmHg (depending on the complications) or S_O_2_ ≤88% (Qaseem et al., 2011). The ACP does not recommend a target to which S_O_2_ should be restored, but there are British guidelines for 88–92% (NICE, 2016). An increase in S_O_2_ of 87–90% corresponds to a ∼4 mmHg increase in arterial P_O_2__, which is about three-times the predicted changes in ${\text{P}}_{O_{2}}^{\prime }$ predicted for 2300 for the A2 scenario (Table 2). The drop in ${\text{P}}_{O_{2}}^{\prime }$ thus cannot have a significant impact on the recommended threshold for supplemental O_2_ usage.

High altitude studies provide another way to consider the physiological consequences of global changes in O_2_. Patients with emphysema living at 7,000 feet ASL show increased cardiovascular complications compared to similar patients living only 4,500 feet ASL (Moore et al., 1982). Also, the patients living at 2,133 m ASL (7,000 ft ASL) died at 68.1 years on average versus 70.1 years for the patients living at 1,372 m ASL (4,500 ft ASL). However, the decrease in PIO_2_ between these altitudes is from 126 to 115 mmHg, which is over sevenfold greater than the decrease in P_O_2__ predicted at with global change by 2300 for the A2 scenario. If age at death scaled similarly with PIO_2_ at sea level, the change in life expectancy for year 2300 would be a few months. This is an upper bound to impacts because individuals at sea level are less sensitive to changes in ${\text{P}}_{O_{2}}^{\prime }$, as discussed above.

Interestingly, almost the same decrease in inspired P_O_2__ was found to be significant for another study of patients with chronic obstructive pulmonary disease (COPD) trying to determine if hypoxia during air travel proposed a risk (Gong et al., 1984). Generally, airplanes are pressurized to maintain an equivalent altitude of no higher than 8000 ft, which maintains arterial O_2_-hemoglobin saturation ≥85%, corresponding to a P_O_2__ of about 55 mmHg, in healthy subjects (De La Zerda and Powell, 2014). In the patients studied by Gong et al. (1984), arterial P_O_2__ fell from a safe level of 55 mmHg at 5000 ft altitude to 50 mmHg at 7,000 ft, a level which requires supplemental O_2_. The difference in inspired P_O_2__ between these altitudes is 124–115, or 9 mmHg, which again is seven times the predicted change in ${\text{P}}_{O_{2}}^{\prime }$ for the A2 scenario. Patients at sea level are almost certainly considerably less sensitive to small changes in ${\text{P}}_{O_{2}}^{\prime }$, which further reinforces the view that the predicted changes in ${\text{P}}_{O_{2}}^{\prime }$ are too small to be significant for these patients.

In considering the effects of global O_2_ decline on disease, it is important to note that changes in arterial P_O_2__ can also exacerbate disease through processes not directly related to O_2_ delivery by the lungs or circulation. In fact, this significance of O_2_ for health and disease was recently recognized with the 2019 Nobel Prize for Physiology or Medicine being awarded to Kaelin, Ratcliffe and Semenza for their discoveries about how cells sense and adapt to O_2_ availability^1^. A key part of their discoveries related to O_2_-sensitive control of gene expression and specifically Hypoxia Inducible Factor-1α (HIF-1α). HIF-1α was identified by Semenza as the transcription factor for the hormone erythropoietein (EPO), which increases red blood cell production in response to hypoxemia. Originally it was a surprise that HIF-1α was expressed in essentially every type of cell tested—including cancer cells—but now this is recognized as a hallmark of the vital central role of O_2_ in homeostasis. Recent studies show that HIF-1α plays an important role in physiological processes as diverse as inflammatory responses (Zinkernagel et al., 2007) and neural plasticity (Moya et al., 2020), illustrating how O_2_ levels affect much more than just mitochondrial function. Hence, it may not be surprising that there appears to be natural selection for O_2_-sensitive transcription factors in high altitude populations as discussed below, although again the magnitude of O_2_ decreases may be greater than the global changes we are considering.

### Reproduction

A major challenge to long-term habitation at high elevation is reduced birth weight impacting reproductive success. In the Rocky Mountains, Moore et al. (1998) found that birth weight decreases by ∼100 g for every km ASL, corresponding to a decrease in inspired P_O_2__ of 13 mmHg. Assuming this relationship also holds at sea level, and assuming P_O_2__ at sea level drops by 2 mmHg, as assumed the most extreme scenario, this would decrease birth weight by ∼15 g relative to a normal birth weight of 2,500 g. This is likely an upper bound because it is based on an extreme estimate of O_2_ loss and because of the non-linearities in O_2_ exchange discussed earlier. The impacts at higher altitude would also be small because predicted P_O_2__ changes are also small (Table 2).

### Evolution

Over geologic time, changes in O_2_ are known to have had a large impact on evolution of mammals and other organisms (Falkowski et al., 2005; Powell, 2010). These evolutionary changes have been in response to fairly large changes in in O_2_, ranging from 60 to 140% of present atmospheric levels.

Hypoxia at high altitude is also known to be a potent force of natural selection in humans. Hundreds of studies published to date provide evidence that Tibetan, Andean, and Ethiopian highland populations have genetic adaptations to high altitude that involve various hypoxia sensing and response genes, including those in the HIF pathway (Simonson, 2015; Moore, 2017). The genomes of Tibetan individuals exhibit adaptive signatures at several genes, including *EPAS1*, which encodes the α subunit of the HIF-2 transcription factor (HIF-2α), and *EGLN1*, prolyl hydroxylase 2 (PHD2) that targets HIF-α subunits for degradation under conditions of normoxia. Both are associated with relatively lower hemoglobin concentration in Tibetans resident at high altitude (Beall et al., 2010; Simonson et al., 2010; Yi et al., 2010). The genomes from Andean and Ethiopian highlanders also exhibit various adaptive signals, including some of the same gene regions identified in Tibetans, although the precise adaptive changes appear to be distinct. For example, functional variants identified at the *EGLN1* locus in Tibetans are absent or found at low frequency in Andeans (Heinrich et al., 2019). The distinct population histories of continental populations (e.g., different genetic backgrounds, admixture events, and generations at altitude) are important factors that contribute to variation across and even within continental populations (Wuren et al., 2014; Simonson, 2015).

It seems possible that natural selection of populations at higher elevations is still ongoing. What is clear from our analysis, however, is that this trajectory cannot be significantly modified by the impacts of fossil-fuel burning on atmospheric O_2_, because these changes are tiny compared to even modest changes in elevation (Table 2).

### Cognition

Brain function is critically sensitive to O_2_ supply, as the brain typically consumes about 20% of the body’s O_2_ uptake while comprising only 2% of body mass (Guyton and Hall, 1996; West, 2016). Brain impairment on exposure to high altitude has been demonstrated for both short-term and long-term exposure, including during childhood development (West, 2017). It should be noted, however, that our understanding of cognitive impacts of long-term exposure to low ${\text{P}}_{O_{2}}^{\prime }$ is informed by very few studies. Childhood development studies are especially difficult because they require comparing one group of individuals to another group, with potential confounding influences of cultural and environmental factors. Significant effects have been resolved only for large changes in altitude. The studies on young adults by Yan et al. (2011), for example, involve comparing a group raised between 2,400 and 4,200 m, with a control group raised below 400 m. These groups were thus exposed to changes in ${\text{P}}_{O_{2}}^{\prime }$ that are 25–100 times larger than the predicted drop in ${\text{P}}_{O_{2}}^{\prime }$ in sea level by year 2300. Because the future losses of O_2_ are equivalent to such small changes in altitude, we conclude that any cognitive impacts of this loss can be at most of very minor significance.

## Discussion

From measurements and models it is well established that the background level of O_2_ in the atmosphere is declining slowly. The decline is mostly caused by fossil-fuel burning with smaller impacts from changes in ocean and land biogeochemistry. These processes are estimated to have caused the O_2_ partial pressure at sea level to drop by ∼0.21 mmHg from a preindustrial times to 2018 from a baseline of 159 mmHg. Based on results presented here, we estimate that, if no steps are taken to mitigate continued exploitation of fossil-fuels, O_2_ will continue to decline, eventually dropping by 1.3 mmHg over a several hundred-year time frame. If humans additionally exploit resources comprising currently uneconomic or undiscovered fossil fuels, the ultimate decrease might amount to 2.5 mmHg many centuries into the future. In either of these very extreme scenarios, atmospheric CO_2_ will rise above 1,200 ppm, associated with global warming of 5°C or more, triggering climate changes considered “beyond catastrophic” (Xu and Ramanathan, 2017). In a less extreme scenario, in which dependence on fossil-fuels is reduced before reserves are exhausted, capping the CO_2_ rise to ∼550 ppm, the O_2_ drop will likely be around 0.5 mmHg. Warming would then be reduced to ∼3°C, which is still considered catastrophic (Xu and Ramanathan, 2017). More aggressive measures to curtail fossil-fuel emissions and warming would likely lead to even smaller O_2_ changes. Above sea level, the declines in O_2_ partial pressure will be smaller than at sea level, due to an offsetting increase in barometric pressure from warming. In fact, above ∼3,000 m ASL, O_2_ partial pressure to is expected slightly increase, despite O_2_ loss globally.

Relatively small changes in altitude or barometric pressure are required to produce similar changes in O_2_ partial pressure. In the high-end A2 scenario considered here, a person living at sea level in year 2300 will experience an O_2_ partial pressure similar to a person living at an altitude of 70 m ASL today. A person living at 4,000 m altitude in year 2300 will experience a O_2_ partial pressure similar to someone living at ∼3,992 m today.

We have considered the possible impacts of these small O_2_ changes on human health, focusing on normal human function, athletic performance, disease, human reproduction, evolution, and cognition. In no case do we find that the changes are significant enough to raise concerns.

These conclusions are based on changes in O_2_ partial pressure in a clean outdoor setting, but a consideration of the additional impacts of O_2_ changes due to local combustion or respiration within an indoor or urban setting does not alter our conclusions. The OSHA occupational exposure limit for indoor CO_2_ is 5,000 ppm,^2^ which compares to the natural background of ∼400 ppm. To raise the CO_2_ concentration by respiration within a confined space from 400 to 5,000 pm, yields a drop ${\text{P}}_{O_{2}}^{\prime }$ of ∼ (0.005 - 0.0004) × 760 = ∼ 4 mmHg at sea level, which is similar to the drop that occurs on ascent of ∼220 m. Typical indoor CO_2_ exposures are <∼1,400 ppm (Karnauskas et al., 2020), so this limit in fact is rarely achieved. As background O_2_ partial pressures drop in the future, indoor partial pressures will drop in parallel from their lower starting point. Our purpose is not to analyze the physiological impact of a ∼4 mmHg indoor O_2_ loss (though we expect it is very small). But clearly the incremental impact of an additional loss over the next few centuries of 1.3 mmHg, for example, will be very minor.

The ongoing changes in atmospheric O_2_ could also potentially impact other aerobic life, such as animals, plants, fungi, bacteria, etc., a topic which remains to be carefully reviewed. We note that even in oxygen-starved aquatic systems, however, the impacts will generally be small because the low O_2_ levels in these systems are controlled by processes below the water surface, such as solubility, sluggish circulation and high inputs of detritus. Tiny changes in the atmospheric boundary condition will not normally have much impact on these systems. Rather, the specter of large future expansion of ocean suboxic zones with dire consequences for ocean life rests firmly on the effects of global warming itself by way of reduced O_2_ solubility and ocean circulation change (Shaffer et al., 2009; Oschlies et al., 2018).

The O_2_ loss over the next few centuries will be dominated by fossil-fuel burning. The ultimate potential loss is capped by known reserves of fossil-fuel, and future trajectory will be largely governed by actual usage trajectories. The model used here predicts that O_2_ loss from fossil-fuel burning will be offset at the ∼30% level by O_2_ sources from the land and ocean. The land source is driven by fertilization from rising CO_2_ leading to accumulation of land biomass. The ocean source is driven by warming-induced solubility changes of O_2_ in seawater and by increased ocean stratification, which increases atmospheric O_2_ at the expense of greater deficits of dissolved O_2_ in the ocean interior. These processes are known to be offsetting O_2_ losses today, and the model is credible because it accounts well for changes in O_2_ to date. Future predictions of these biogeochemical responses are clearly speculative, but they are in any case of secondary importance. We find no basis to support the higher estimate of O_2_ loss from Livina et al. (2015) which is based on mathematical extrapolation without geochemical underpinning.

In theory, O_2_ loss could be exacerbated by strategies to reduce CO_2_ emissions, such as carbon capture and storage (CCS), which captures CO_2_ and stores it underground. CCS offsets the CO_2_ emission from fossil-fuel burning and the associated global warming, but does not offset the O_2_ loss because the captured carbon is in the form of CO_2_, rather than biomass. Although the model simulations presented here did not consider CCS, it is easy to set bounds on O_2_ by considering an extreme version of the A2 scenario in which all the CO_2_ eventually released from fossil-fuel burning is captured and stored. Because this eliminates warming and CO_2_ buildup, the O_2_ loss is governed by fossil-fuel usage alone, yielding an ultimate drop of 1.8 mmHg or 1.1% of the initial pressure of 159 mm Hg. This fractional change would be felt throughout the atmospheric column, equivalent to a nearly uniform upwards shift in altitude of ∼90 m. The societal benefit of this outcome in terms of climate mitigation would greatly outweigh any small impacts from the changes in O_2_. This merely illustrates that concern over O_2_ loss is a very weak argument against CCS as a mitigation strategy.

More generally, there is now clearly an urgent need to curtail fossil-fuel CO_2_ emissions to avoid catastrophic climate change, which will certainly dominate the impact on human health. The corresponding loss of O_2_, which is of biogeochemical interest, is merely a physiological curiosity in comparison. The case for action rests on the consequences for CO_2_, not O_2_.

## Author Contributions

RK and FP drafted the manuscript with contributions from other coauthors. RK contributed measurements of O_2_/N_2_ ratio for Figure 1 and carried out calculations of pressure O_2_ changes at higher elevations. GS contributed published model runs in support of Figures 2, 3. All authors contributed to the ideas and framing.

## Conflict of Interest

The authors declare that the research was conducted in the absence of any commercial or financial relationships that could be construed as a potential conflict of interest.

## Footnotes

1 https://www.nobelprize.org/prizes/medicine/2019/summary/

2 https://www.osha.gov/dsg/annotated-pels/tablez-1.html

## Appendix A. O_2_ Units

### Total Atmospheric O_2_ Inventory

This can be expressed in moles or mass of O_2_. Using the O_2_ mole fraction of 0.2094 from Tohjima et al. (2005) for year 2000, a corresponding molecular weight of dry air of 28.96 g/mol, and the total mass of dry air of 5.135 × 10^18^ kg (Trenberth and Smith, 2005) yields an O_2_ inventory of 3.7125 × 10^19^ moles or 37125 Pmol.

### O_2_ Mole Fraction in Dry Air, X_O_2_

This unit express the ratio of moles of O_2_ per mole of dry air in a sample, often loosely called “concentration” and typically expressed as percent or in ppm.

### O_2_ Partial Pressure, P_O_2__, ${\text{P}}_{O_{2}}^{\prime }$

This unit is used to describe O_2_ tension in blood, for example, which is relevant to assess O_2_ transport to vital tissues. Typical units are atmospheres, or mmHg, Torr or kPa for physiology. For physiological applications, it is useful to quantity ambient P_O_2__ in relation to air with the same pressure but zero humidity, which is given by ${\text{P}}_{O_{2}}^{\prime }$ = X_O_2_ × P_tot_, where P_tot_ is the total barometric pressure (including water vapor), and X_O_2_ is the O_2_ dry air mole fraction. At sea level and 1 standard atmosphere this yields ${\text{P}}_{O_{2}}^{\prime }$ = (0.2094)(760 mmHg) = 159.1 mmHg.

### Inspired O_2_ Partial Pressure, P_IO_2_

Another physiologically relevant measure of the ambient O_2_ is the partial pressure in the lung airways, which is lower than ${\text{P}}_{O_{2}}^{\prime }$ due to dilution by water vapor (as X_O_2_ and P_tot_ are unchanged). This quantity is known as the inspired O_2_ partial pressure, given by P_IO_2_ = X_O_2_ × (P_tot_ – P_sat_(37)) = ${\text{P}}_{O_{2}}^{\prime }$ – X_O_2_⋅P_sat_(37), where P_sat_(37) = 47 mmHg is the saturation vapor pressure of water at body temperature 37°C and X_O_2_ × Psat(37) = 10 mmHg.

### Present Atmospheric Level (PAL)

This unit is used in the geochemistry community to express the abundance of O_2_ in the atmosphere in the distant past. The unit measures the total O_2_ abundance level in the atmosphere in relation to the present 21% level. An atmosphere containing 1.1 PAL has 10% more O_2_ molecules or 10% more O_2_ mass than our current atmosphere.

### δ(O_2_/N_2_)

This is the conventional unit for reporting changes in atmospheric O_2_ abundance in the modern atmosphere. This unit expresses the relative deviation in the molar O_2_/N_2_ ratio from a standard value of this ratio:

$$\delta \,\left({\text{O}}_{2}/{\text{N}}_{2}\right)=\left({\left({\text{O}}_{2}/N_{2}\right)}_{\text{sample}}-{\left({\text{O}}_{2}/{\text{N}}_{2}\right)}_{\mathrm{standard}}\right)/{\left({\text{O}}_{2}/{\text{N}}_{2}\right)}_{\text{standard}}$$

δ(O*_2_*/N*_2_*) is typically multiplied by 10^6^ and expressed in “per meg units,” where 1 per meg = 0.0001%. The O_2_/N_2_ ratio can be influenced both by changes in O_2_ or N_2_, but changes in N_2_ are typically much smaller.

### Example Calculation

We start with an idealized well-mixed dry atmosphere that initially contains 21% O_2_, 78% N_2_, and 1% Ar. These figures refer to mol%. We assume the sum of the partial pressure of these gases equals 1 atm at sea level. We assume ideal gas behavior. The sea-level partial pressure of O_2_ is thus 0.21 atm, the dry air O_2_ mole fraction is 21% and the O_2_/N_2_ ratio is 21/78, which is taken as the standard ratio. The initial value of δ(O*_2_*/N*_2_*) is thus 0% or 0 per meg.

Suppose that 1% of the O_2_ content of the atmosphere is removed without any changes in N_2_ or Ar. With these assumptions, the final PAL level is 0.99 and the final O_2_/N_2_ ratio value is 0.99 × 21/79 corresponding to δ(O_2_/N_2_) = -1% or -10,000 per meg. The final dry air O_2_ mole fraction is 0.99 × 0.21/(0.79 + 0.99 × 0.21) = 0.208338, where the denominator (0.79 + 0.99 × 0.21) accounts for the decrease in total moles caused by the O_2_ loss. If CO_2_ is produced in association with the O_2_ loss, this would tend to offset the changes in the denominator. The final O_2_ partial pressure is given to a good approximation by 0.99 × 0.21 = 0.2079 atm, which assumes that total barometric pressure drops in proportion to total moles. Some minor limitations to this assumption are discussed in Appendix B.

### Unit Conversion Factors

The above considerations suggest these conversion factors for global changes at sea level: 1 PAL = 37125 Pmol O_2_ = 10^6^ per meg (δ(O_2_/N_2_)) = 159.1 mmHg (dry air partial pressure, ${\text{P}}_{O_{2}}^{\prime }$). Conversion based on these factors may be in error by a few percent depending also on changes in N_2_, CO_2_, and H_2_O, as discussed in Appendix B.

### Appendix B. on the Proportionality Between ${\text{P}}_{O_{2}}^{\prime }$ and Global O_2_ Inventory

Figure 3 computes changes in ${\text{P}}_{O_{2}}^{\prime }$ from the results of Shaffer et al. (2009) on the assumption that ${\text{P}}_{O_{2}}^{\prime }$ is proportional to the total O_2_ inventory. Here we identify several corrections to this assumption, showing they are all small.

To relate ${\text{P}}_{O_{2}}^{\prime }$ to the composition of the atmosphere, we take

(B1) $$P_{O_{2}}^{\prime }={\text{X}}_{O_{2}}\times {\text{P}}_{\text{tot}}$$

(B2) $${\text{X}}_{O_{2}}={\text{N}}_{O_{2}}/\left({\text{N}}_{N_{2}}+{\text{N}}_{O_{2}}+{\text{N}}_{\text{Ar}}+{\text{N}}_{{\mathrm{CO}}_{2}}+\ldots \right)$$

(B3) $${\text{P}}_{\text{tot}}=\left(\text{g}/\text{A}\right)\cdot \left({\text{N}}_{N_{2}}\cdot {\text{m}}_{N_{2}}+{\text{N}}_{O_{2}}\cdot {\text{m}}_{O_{2}}+{\text{N}}_{\text{Ar}}\cdot {\text{m}}_{\text{Ar}}+{\text{N}}_{H_{2}\,O}\cdot {\text{m}}_{H_{2}\,O}+{\text{N}}_{{\mathrm{CO}}_{2}}\cdot {\text{m}}_{{\mathrm{CO}}_{2}}+\ldots \right)$$

(B4) $${\text{W}}_{O_{2}}={\text{N}}_{O_{2}}\times {\text{m}}_{O_{2}}/\left({\text{N}}_{N_{2}}\cdot {\text{m}}_{N_{2}}+{\text{N}}_{O_{2}}\cdot {\text{m}}_{O_{2}}+{\text{N}}_{\text{Ar}}\cdot {\text{m}}_{\text{Ar}}+{\text{N}}_{H_{2}\,O}\cdot {\text{m}}_{H_{2}\,O}+{\text{N}}_{{\mathrm{CO}}_{2}}\cdot {\text{m}}_{{\mathrm{CO}}_{2}}+\ldots \right)$$

where N_*i*_ and m_*i*_ are the number of moles and molecular mass of species i, X_O_2_ is the dry air O_2_ mole fraction (0.2094), W_O_2_ is the O_2_ mass fraction of the entire atmosphere (∼0.2314), P_tot_ is barometric pressure at sea level, g is gravitational acceleration, and A is Earth’s surface area. The sums in B2–B4 implicitly include contributions from all other trace gases in dry air. The sums in B3 and B4 also include the contribution from water vapor but the sum B2 for dry air does not.

To evaluate the dependence of ${\text{P}}_{O_{2}}^{\prime }$ on O_2_ amount, we take the logarithmic derivative of Eq. B1 with respect to N_O_2_ with substitutions from B2 to B4, yielding

(A5) $${\text{dP}}_{{\text{O}}_{2}}^{{}^{\prime }}/{\text{P}}_{{\text{O}}_{2}}^{{}^{\prime }}=\left(1+{\text{W}}_{{\text{O}}_{2}}-{\text{X}}_{{\text{O}}_{2}}\right)\,\delta \,N_{{\text{O}}_{2}}/{\text{N}}_{{\text{O}}_{2}}\approx 1.022\,\delta \,N_{{\text{O}}_{2}}/{\text{N}}_{{\text{O}}_{2}}$$

The assumption that ${\text{P}}_{O_{2}}^{\prime }$ is proportional to N_O_2_ thus underestimates changes in ${\text{P}}_{O_{2}}^{\prime }$ by 2.2%. This small correction arises because barometric pressure at sea level does not scale in proportion to the number of moles of air (as would be true for an ideal gas in a constant volume) but rather with atmospheric mass. O_2_ has a molecular weight (32) that exceeds the average molecular weight of air (29) and thus makes a proportionally larger contribution to total mass than total moles.

${\text{P}}_{O_{2}}^{\prime }$ is also sensitive to changes in the abundance of CO_2_, via the presence of N_CO_2_ in equations B2 and B3. Taking the logarithmic derivative of Eq. B1 with respect to N_CO_2_ with substitutions from B2 to B4 yields

(A6) $${\text{dP}}_{{\text{O}}_{2}}^{{}^{\prime }}/{\text{P}}_{{\text{O}}_{2}}^{{}^{\prime }}=\left({\text{W}}_{{\text{CO}}_{2}}-{\text{X}}_{{\text{CO}}_{2}}\right)\,\delta \,N_{{\text{CO}}_{2}}/{\text{N}}_{{\text{CO}}_{2}}\approx \left(\left({\text{W}}_{{\text{CO}}_{2}}-{\text{X}}_{{\text{CO}}_{2}}\right)/{\text{X}}_{{\text{CO}}_{2}}\right)\,\delta \,X_{{\text{CO}}_{2}}=0.516\,\delta \,X_{{\text{CO}}_{2}}$$

where W_CO_2_ and X_CO_2_ are the weight and mole fractions of CO_2_ in air, defined similarly to B2 and B4. Assuming CO_2_ eventually rises to from a preindustrial level of 280 ppm to 1000 ppm (per A2 in Figure 3), this yields dP${}_{O_{2}}^{\prime }$ = (159)(0.516)(0.00100-0.000280) = 0.06 mmHg, which is negligible.

Atmospheric water vapor is also expected to increase with climate warming. Water vapor has no impact on the dry air mole fraction X_O_2_ but comprises 0.25% of the mass of the atmosphere (Trenberth and Smith, 2005), and thus contributes 0.25% to P_tot_ in Eq. B1. Assuming that the water content scales with warming according to the saturation vapor curve, and adopting warming estimate of 5°C from the A2 scenario (Figure 3), this yields an increase in water vapor of ∼40%. This causes barometric pressure to increase by 0.1%, corresponding to an increase in ${\text{P}}_{O_{2}}^{\prime }$ at sea level of 0.001 × (159 mmHg) = 0.16 mmHg. This compares to the drop in ${\text{P}}_{O_{2}}^{\prime }$ of 1.4 mmHg estimated under the A2 scenario due to global O_2_ loss (Figure 3) at year 2150, when Earth has warmed by 5C.

Warming causes the oceans to release N_2_ to the atmosphere due to the temperature dependence of the N_2_ solubility in seawater. Keeling et al. (2004) estimate a sensitivity of ∼2 × 10^–6^ of the atmospheric N_2_ inventory per 100 ZJ of ocean warming. Assuming the oceans as a whole eventually warm by an average of ∼4°C for the A2 scenario (e.g., Shaffer et al., 2009), this yields a relative increase in atmospheric N_2_ inventory of 0.04%, which in turn impacts both X_O_2_ and P_tot_ at the 0.04% level but in opposite directions. The impact on ${\text{P}}_{O_{2}}^{\prime }$ via Eq. B1 is less than 0.01% because the contributions from X_O_2_ and P_tot_ nearly cancel, and is thus completely negligible.

In summary, we have quantified several small corrections required to the estimated changes in ${\text{P}}_{O_{2}}^{\prime }$ in Figure 3. One correction results from changes in molecular weight of dry air with O_2_ loss, leading to a ∼2% correction in the direction of amplifying the ${\text{P}}_{O_{2}}^{\prime }$ decrease. A second and larger correction results from an increase in atmospheric water vapor which increases ${\text{P}}_{O_{2}}^{\prime }$ through the impact on barometric pressure. In the context of the future scenarios, this water vapor impact amounts to ∼12% of the calculated decrease in ${\text{P}}_{O_{2}}^{\prime }$ due to O_2_ loss, partially offsetting the overall ${\text{P}}_{O_{2}}^{\prime }$ decrease. The combined impact of both corrections is of order ∼10% in the direction of offsetting the ${\text{P}}_{O_{2}}^{\prime }$ decrease. Adjusting our calculation for these small effects would not change any conclusions. Other corrections due to changes in CO_2_ and N_2_ are even smaller.

### Appendix C. Shaffer et al. (2009) Model Details

The A2 and B1 scenarios used in Shaffer et al. (2009) prescribe future fossil-fuel burning and land-use change, which drive CO_2_ emissions and O_2_ loss. The scenarios ascribe minor importance to land-use change emissions going forward. The intermediate-complexity carbon/climate model used in Shaffer et al. (2009) computes changes in CO_2_ and global temperature which drive additional changes in O_2_ from changes in ocean and land biogeochemistry (Shaffer et al., 2008). Shaffer et al. (2009) extend these scenarios past year 2100 using polynomial functions that taper emissions gradually toward zero around 2200. The A2 and B1 scenarios are often considered to bracket possible outcomes: the A2 scenario allows for emissions growth without any mitigation (“business as usual”), while B1 assumes emissions are partly curtailed to mitigate climate changes. The model assumes O_2_:C stoichiometric ratios of 1.391 for fossil-fuel burning and 1.1 for land biospheric exchanges.

Shaffer et al. (2009) report O_2_ changes in units of partial pressure, which they calculate by multiplying the estimated preindustrial sea level O_2_ partial pressure of 0.2094 atm by the relative change in the total atmospheric O_2_ inventory from their model. From the considerations Appendix B, we interpret these results as corresponding to ${\text{P}}_{O_{2}}^{\prime }$.

The predictions shown in Figure 3 used a prescribed climate sensitivity of 3°C per doubling of CO_2_. Shaffer et al. (2009) also carried out simulations with a higher sensitivity of 4.8°C. The choice of climate sensitivity has very little impact on decreases in ${\text{P}}_{O_{2}}^{\prime }$ at sea level because this sensitivity impacts only the land and ocean O_2_ sources and not the O_2_ losses from fossil-fuels and land-use. The changes in ${\text{P}}_{O_{2}}^{\prime }$ at higher altitudes are somewhat sensitive to the assumed climate sensitivity. Higher climate sensitivity causes more warming and therefore larger increases in barometric pressure and ${\text{P}}_{O_{2}}^{\prime }$ at higher altitude while also lowering the cross-over altitude.
